# An electronic diabetes foot risk calculator

**DOI:** 10.1186/1757-1146-6-S1-P15

**Published:** 2013-05-31

**Authors:** Deborah Schoen, David Glance, Sandeep Aggarwal, Sandra Thompson

**Affiliations:** 1 Combined University Centre for Rural Health (CUCRH), University of Western Australia, Perth, WA, 6009, Australia; 2 Centre for Software Practice, University of Western Australia, Perth, WA, 6009, Australia

## Background

The MMEx Diabetes Foot risk calculator was designed in response to the WA Department of Health’s High Risk Foot Model of Care to improve the systematic approach to screening and treatment for high risk foot complications and the 2011 National Health and Medical Research Council’s (NHMRC) National Evidence-Based Guideline on Prevention, Identification and Management of Foot Complications in Diabetes that state, “a solution to the current impasse on the integration of decision support tools into medical software is needed urgently” and “any trained health professional can complete a foot risk stratification”. Teaching foot assessments is straightforward, but practitioners have difficulty with risk stratification and deciding appropriate follow-up and referral.

## Methods

The system has been developed as a collaboration between the University of Western Australia’s Combined Universities Centre for Rural Health and the Centre for Software Practice. The software design was based on the evidence-based foot risk stratification of the NHMRC and incorporated a risk calculator, or electronic decision support, as currently used in cardiovascular risk assessment.

## Results

The MMEx Diabetes Foot risk calculator is modified based upon input from a number of service providers from different disciplines. It is now available in MMEx, a clinical information system for primary care, or from the App Store.

## Conclusion

The calculator has translated clinically relevant research into practice enabling a systematic approach to Medicare Enhanced Primary Care plans, which includes patients receiving foot risk stratification at their first visit. It can improve time management, Medicare reporting requirements, clinical planning and communication.

